# Supplementary material for: TriTrypDB: An integrated functional genomics resource for kinetoplastida
Source: PLoS Negl Trop Dis. 2023 Jan 19;17(1):e0011058. doi: 10.1371/journal.pntd.0011058 (PMC9888696; doi:10.1371/journal.pntd.0011058)
Supplement: S1 File — Fig A Overall trend of genomes and other functional datasets available in TriTrypDB between release 1.0 (October 2009) and release 59 (October 2022). Fig B Accessing record pages of popset isolate sequences by conducting a dedicated search from the home page. Fig C The Gene Ontology terms table from the gene pages. An example from gene Tb927.8.4470 (chaperone protein DnaJ, putative, J40) showcasing annotations from multiple sources such as GeneDB, UniProt and TrypTag databases. The descriptions of data available in the different columns of this GO terms table are also provided here. (DOCX) [file pntd.0011058.s001.docx]

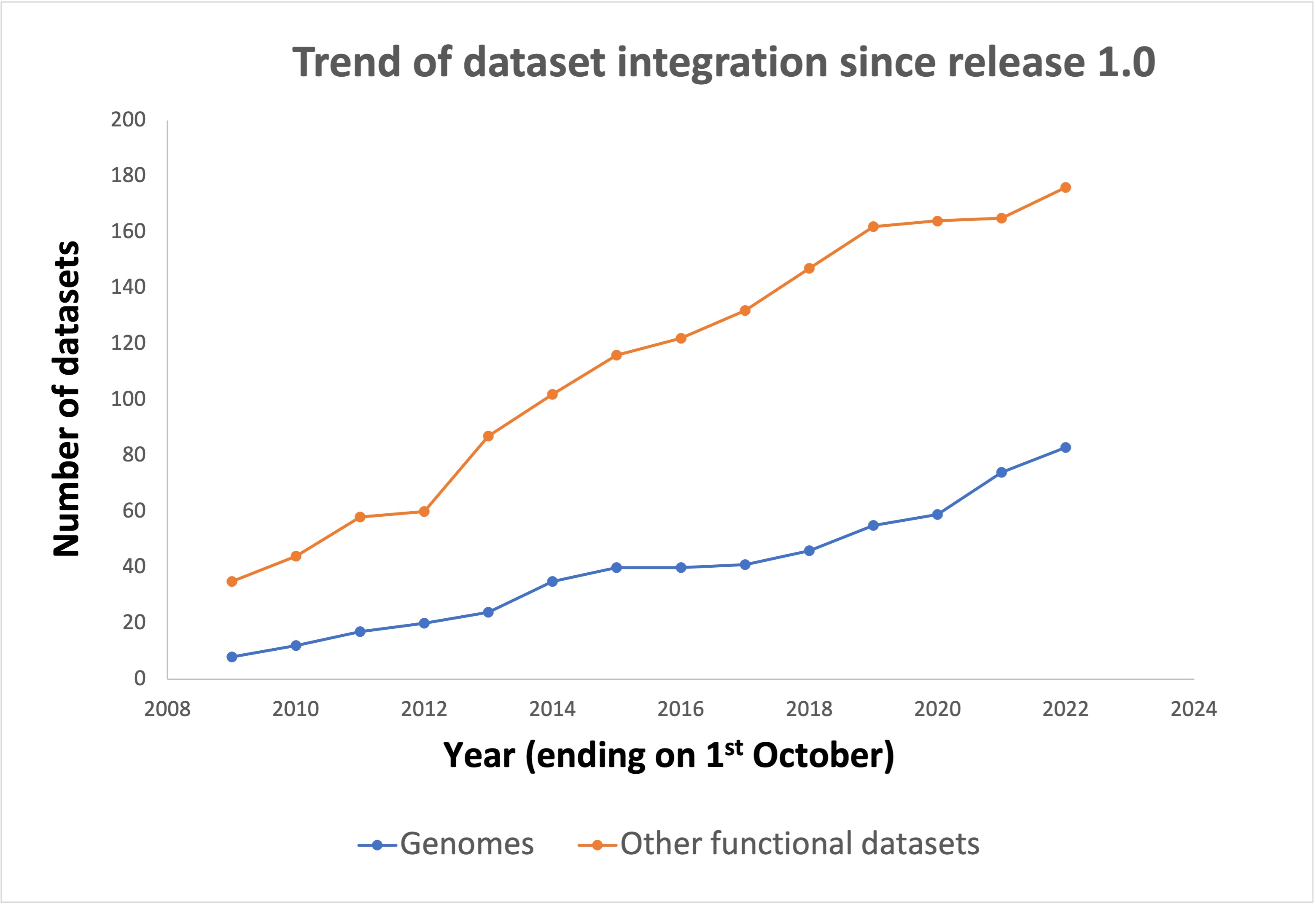


**Fig** **A** Overall trend of genomes and other functional datasets available in TriTrypDB between release 1.0 (October 2009) and release 59 (October 2022).


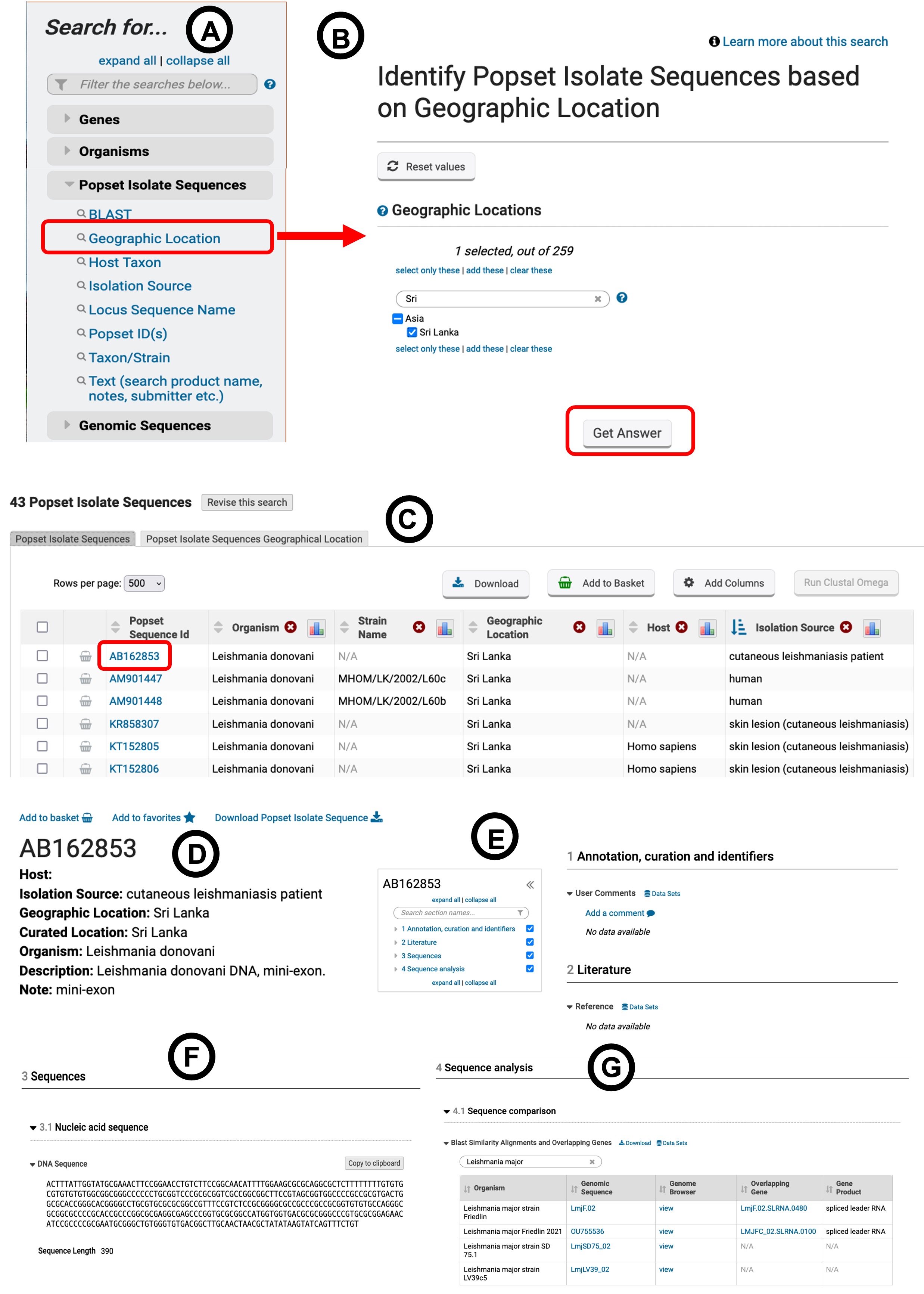


**Fig B** Accessing record pages of popset isolate sequences by conducting a dedicated search from the home page.

(A) The left hand search panel from the homepage showing searches under the Popset isolate sequences. The search for sequences on the basis of isolated ‘geographic location’ is highlighted (red box). (B) The search page for retrieval of sequences from chosen geographical locations. After choosing geographical locations (e.g. Sri Lanka), click the “Get Answer” button. (C) The results page showing retrieved sequences with available metadata. By clicking on a sequence ID (highlighted red box), users can access the record page for the chosen popset isolate sequence. (D) The header section showing key details of the retrieved sequence. (E) The collapsible contents menu at the left showing the different categories of available data and the first two out of four sections of compiled data. (F) ‘Sequences’ section displaying the actual isolate sequence. (G). ‘Sequence analysis’ section displaying the genomic sequences and overlapping genes that are retrieved by BLAST alignment to this isolate sequence. The results are filtered to show only the aligned sequences from the different strains of *Leishmania major*.


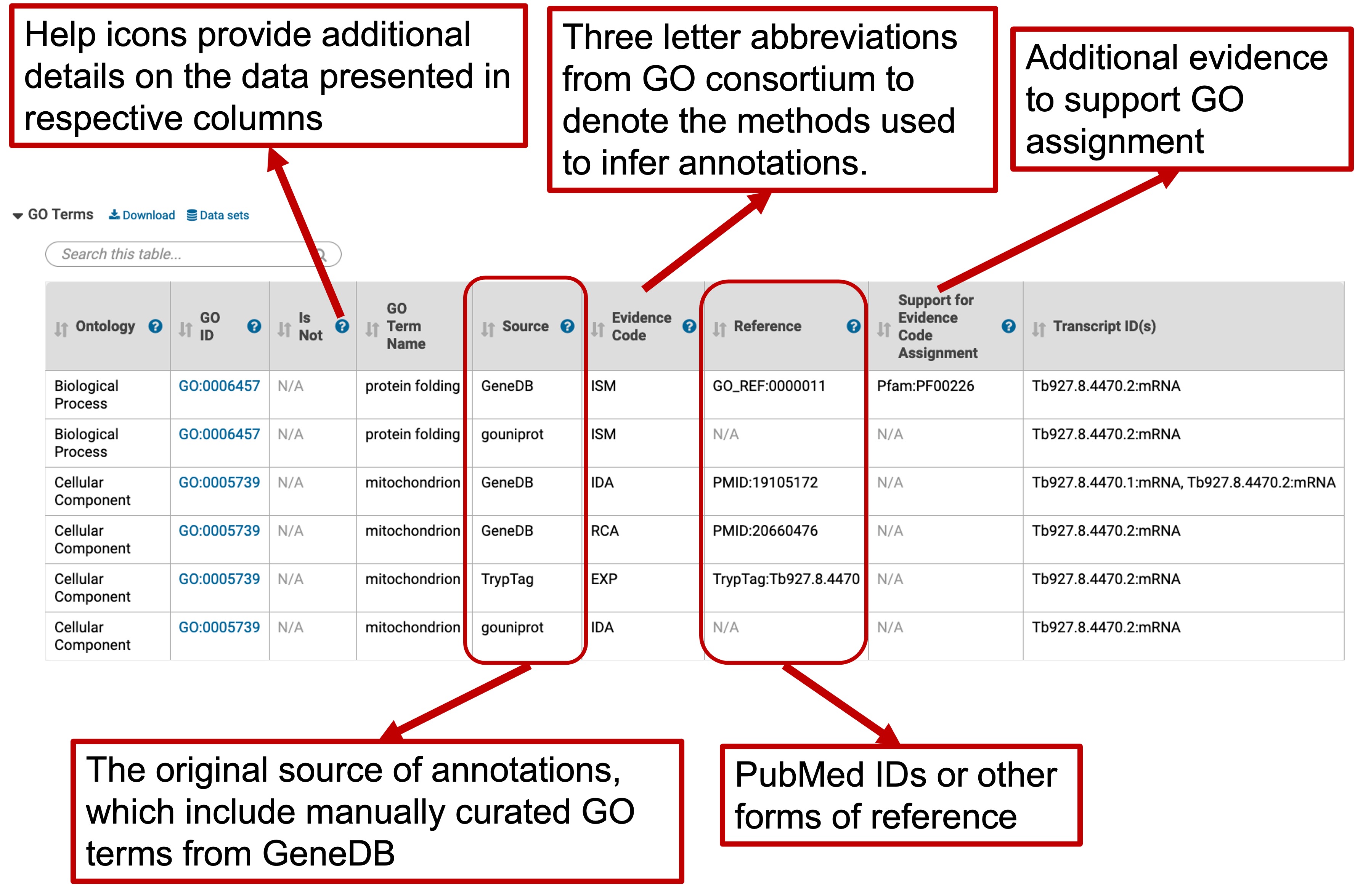


**Fig C The Gene Ontology terms table from the gene pages.**

An example from gene Tb927.8.4470 (chaperone protein DnaJ, putative, J40) showcasing annotations from multiple sources such as GeneDB, UniProt and TrypTag databases. The descriptions of data available in the different columns of this GO terms table are also provided here.
