# Supplementary material for: TriTrypDB: An integrated functional genomics resource for kinetoplastida
Source: PLoS Negl Trop Dis. 2023 Jan 19;17(1):e0011058. doi: 10.1371/journal.pntd.0011058 (PMC9888696; doi:10.1371/journal.pntd.0011058)
Supplement: Attachment — Submitted filename: TriTrypDB_manuscript_reviewer_responses_v2.docx [file pntd.0011058.s002.docx]

December 5^th^, 2022

The manuscript (PNTD-D-22-01301R1) was returned to us on 1^st^ Dec 2022, with some extra formatting and queries over copyright of logos and screenshots.

By email, we resolved that there was no issue over copyrights and logos, since we are the copyright holders for the TriTrypDB website, it is fully open access and compliant with sharing under CC-BY.

In this upload, we also change pdf versions of the figures to high-resolution tiffs, added section headers for Methods and Results, and added one initial to an author name, to tally with the submission form.

Other responses to reviewers’ queries are below,

Best wishes

Andy Jones

******

November 28, 2022

Dr João L. Reis-Cunha & Dr Ricardo Fujiwara

PLoS Neglected Tropical Diseases

Dear Drs Reis-Cunha & Fujiwara,

Thank you very much to the editorial team and to the independent reviewers for kindly reviewing our manuscript titled “TriTrypDB: an integrated functional genomics resource for kinetoplastida” for publication in PLoS Neglected Tropical Diseases. We are delighted to hear that you were happy to accept our manuscript for publication subject to modification of the manuscript in accordance with the reviewer recommendations. We are happy to make a resubmission of the manuscript by accommodating those suggestions. Please find below our responses to reviewer comments, which includes information on how we updated the manuscript with reviewer comments.

Thank you very much for considering our manuscript for publication. We are looking forward to hearing from you again.

Yours sincerely,

Andy Jones

***********************

Reviewer's Responses to Questions

<b>Key Review Criteria Required for Acceptance?</b></br></br>
As you describe the new analyses required for acceptance, please consider the following:</br></br>

<b>Methods</b></br></br>
-Are the objectives of the study clearly articulated with a clear testable hypothesis stated?</br>
-Is the study design appropriate to address the stated objectives?</br>
-Is the population clearly described and appropriate for the hypothesis being tested?</br>
-Is the sample size sufficient to ensure adequate power to address the hypothesis being tested?</br>
-Were correct statistical analysis used to support conclusions?</br>
-Are there concerns about ethical or regulatory requirements being met?</br></br>

Reviewer #1: (No Response)

Reviewer #2: This describes a database resource, so this section is not particularly relevant. Please see my general comments below.

Reviewer #3: (No Response)

--------------------

<b>Results</b></br></br>
-Does the analysis presented match the analysis plan?</br>
-Are the results clearly and completely presented?</br>
-Are the figures (Tables, Images) of sufficient quality for clarity?</br></br>

Reviewer #1: (No Response)

Reviewer #2: Again, this paper describes a database resource, so this section is not particularly relevant. Please see my general comments below.

Reviewer #3: (No Response)

--------------------

<b>Conclusions</b></br></br>
-Are the conclusions supported by the data presented?</br>
-Are the limitations of analysis clearly described?</br>
-Do the authors discuss how these data can be helpful to advance our understanding of the topic under study?</br>
-Is public health relevance addressed?</br></br>

Reviewer #1: (No Response)

Reviewer #2: This paper describes TriTrypDB, a sub-component of the larger VEuPathDB project, which is an excellent and vital resource for research into these neglected tropical pathogens. The paper describes the great range of primary and derived data resources, the powerful tools for searching/interrogating them, and the diverse range of work they support. If anything, I feel the impressive first discussion paragraph undersells the fundamental importance of this resource to the field!

**Response:** Thank you very much to the reviewer for all the positive comments regarding TriTrypDB database and the current manuscript.

Reviewer #3: (No Response)

--------------------

<b>Editorial and Data Presentation Modifications?</b></br><br/>
Use  this section for editorial suggestions as well as relatively minor modifications of existing data that would enhance clarity. If the only modifications needed are minor and/or editorial, you may wish to recommend “Minor Revision” or “Accept”.

Reviewer #1: (No Response)

Reviewer #2: Overall, the quality of the text and figures are high. Some descriptions of the data underlying the TriTrypDB database could, however be a little clearer - see general comments below. Some minor changes to the text should clarify these points.

**Response:** We have added additional details to several sections of the manuscript in response to several general comments made by all the reviewers. We hope that this update will clarify the doubts raised by the reviewers and make the manuscript clearer.

Reviewer #3: (No Response)

--------------------

<b>Summary and General Comments</b></br></br>
Use  this section to provide overall comments, discuss strengths/weaknesses of the study, novelty, significance, general execution and scholarship. You may also include additional comments for the author, including concerns about dual publication, research ethics, or publication ethics. If requesting major revision, please articulate the new experiments that are needed.

**Reviewer #1:** This is a nice overview of the TriTrypDB resource. I have a few minor comments.

**Comment:**
- The only part I found confusing was the section on the Tritryp genomes that used to be hosted in GeneDB: "The large-scale re-annotation and curation of these genomes were made possible by continuous funding from the Wellcome Trust for the TriTrypDB database since 2012. The updated annotations are regularly integrated into TriTrypDB through bimonthly releases. As GeneDB was taken offline last year, TriTrypDB serves as the sole authoritative resource for the annotation of these genomes. " What large scale re-annotation is being referred to? Were they annotated independently or were annotations pulled from GeneDB? Is the sentence about updated annotations referring to before or after GeneDB was taken offline? I think it's great to mention that TriTrypDB is now the sole source, but I was tripped up by the rest of the paragraph.

**Response:**

The large-scale reannotation refers to large-scale updates to gene models (including new gene models, changes to coding sequences, genes made obsolete etc). There were ~9k genes updated in the last decade and these changes are summarised in **Table 2.** These annotations were made by staff from the VEuPathDB/ TriTrypDB project on the GeneDB backend annotation database as a collaborative effort between both databases since 2012 (funded by Wellcome to the VEuPathDB project). This combined effort cannot be considered as pulling out annotations from GeneDB as the annotations were made by VEuPathDB/ TriTrypDB curators themselves. The curation efforts by VEuPathDB/ TriTrypDB still continued after GeneDB was taken offline with functional annotations being updated in the releases in the last year. The text has been updated in the ‘Curation and Annotation’ section (**page 15**) to explain this collaborative effort better.

**Comment:**
- Under Record Pages: I felt that the mention of other TriTrypDB pages was a little superficial. "TriTrypDB also provides pages for other record types including popset isolate sequences, genomic sequences (scaffolds), genomic segments, SNPs, ESTs, metabolic pathways and chemical compounds." As someone who hasn't accessed these pages I wondered what they contained/how they were organized/how you get there.   This might be difficult to do because the pages contain such different types of data, but if possible a bit more information might help a reader who is less familiar with these pages.

**Response:**

The record pages section of the manuscript has been updated with a paragraph containing description of these record types and how they can be accessed using searches (**page 11**). An additional supplementary figure (**S2 Fig**) has also been added to illustrate this.

**Comment:**
- "In addition, TriTrypDB collaborates with the Gene Ontology Consortium to create new terms to represent kinetoplastid biology, particularly cellular components and biological processes. " This is interesting and I wasn't aware of it. Really minor suggestion, but it could be interesting to mention what some of these terms are!

**Response:**

A few examples of newly requested Kinetoplastid-relevant biological process and cellular component terms are now added to the ‘Functional annotation’ sub-section of the manuscript (**page 19**). E.g. reservosome, flagellum attachment zone, procyclogenesis, kinetoplast DNA replication etc.

**Comment:**

Under the record pages section there is a typo: "Gene pages host [a] multitude of data in.."

**Response:** Thank you very much for catching this typo. It is corrected now.

**Reviewer #2:** I have some comments and queries. However, I would like to emphasise that these are intend as constructive criticism and desirable changes on the large scale - perhaps outside of the scope of this paper. The core of this paper is already strong, a clear description of a resource me, my lab, and my field use extensively and daily.

**Response:**

Our sincere thanks to the reviewer for his/ her statement on the extensive use of TriTrypDB by their lab and the field. In addition, we are happy to hear that their intent on several of these comments are suggestions for future changes to the database rather than changes to the current manuscript. However, we have responded to all their comments by making things more clear in our response and also accommodating them in our manuscript.

**Comment:**

I find the description of the impressive range of data/analyses that TriTrypDB enables access to clear. However I am afraid I am left a little confused by what TriTrypDB is NOT responsible for, especially at the foundational level, eg.:
Curated genomes. Is 'ownership' of these limited to hosting the sequence and carrying out annotation, or will efforts be made to improve assembly based on new data?

**Response:**

The ownership of the genomes are limited to updating structural and functional annotations and updating those annotations in the INSDC repositories. We have obtained permissions for this from the providers. We currently do not change the underlying assembly from new data. However, we reach out to the community and host new genome assemblies of already existing organisms. This has now been made clear in the second paragraph of the ‘Curation and Annotation’ section (**pages 15/16**).

**Comment:**
Genome annotation. Does TriTrypDB carry out proactive gene model updates for curated genomes, updated/corrected gene models for newly submitted genomes, de novo annotation of gene models for new genomes, etc. or must these be provided by the community for new genomes?
Overall, this is a question of defined scope and I suggest making distinctions between "owned" sequence data that may change, "hosted" sequence data that will not change and "live" data which is recalculated each release, and to think where else similar distinctions may help for other data types.
I hope that more clarity here would help the community effectively feed data into TriTrypDB and vice versa.

**Response:**

TriTrypDB (in collaboration with GeneDB until last year) carried out proactive gene model updates for curated genomes (as summarised in **Table 2**) and these were updated in our releases. The genomes that are tagged as “curated” are the ones that are updated with expert curation. We do not usually conduct *de novo* annotation for newly sequenced genomes and we obtain both genomes and annotations from providers. However, we also carried out *de novo* annotations when suitable annotation was not available and it was requested by the community (e.g. *Leishmania amazonensis*). This was already mentioned in the structural annotation section of the manuscript (**page 17**). We do not however update gene models for newly submitted genomes or generate new gene models as these activities are beyond our scope due to the large number of genomes we host and funding and staff limitations. We have added a couple of sentences in the structural annotation section to mention this point.

Although we do not expect to curate the vast majority of representative (or reference genomes), these are added to Apollo and hence can be curated by the user communities and official gene models can be updated when significant changes are made. We have also obtained approval from almost all the genome providers (with some exceptions) for co-ownership of the genomes and the updating of genomes in INSDC repositories is planned for the future. This is now added to the second paragraph of the section on ‘Curation and Annotation’ (**pages 15/16**). You can also find details on which genomes are updated in each release from the news section.

**Comment:**
I found it clear that TriTrypDB draws data from NCBI genomes and SRA, however I found the inverse unclear. Eg. Do updates to the reference genome annotations (particularly gene) get fed back to the NCBI gff files for those genomes? Similarly for community-sourced annotation updates for non-reference genomes? Are all genomes on TriTrypDB also in NCBI genomes?
I realise that good open data practice for the authors of any new genome to appropriately deposit the raw and assembled sequence data into appropriate repositories, but this is not always done - particularly (it seems) for gene models. I suspect this has knockon effects, eg. are all predicted protein sequences available to Uniprot, gathered for UniRef, and similar?

**Response:**

All genome assemblies except two are available in INSDC repositories including NCBI. The exceptions are two genomes that we have integrated in the past before the implementation of the current (EBI) analysis pipelines. We are still keeping those genomes due to their value to the community. However, we contacted the genome providers to submit to GenBank in these cases, with no response from the providers. We encourage all our data providers to submit their data to relevant repositories and we do not currently accept genome assemblies that are not submitted to INSDC. But, we still accept annotations directly from the providers (without submissions to INSDC). A sentence has been added at the end of the first paragraph under the updated data processing subsection (**page 8**).

We do not currently submit updated annotations to INSDC (including NCBI). We are currently working with NCBI on devising the mechanism for synchronisation of TriTrypDB/ VEuPathDB records with them and this is on our future plans (please also see response to the question above).

**Comment:**

On a related note, you indicate TriTrypDB now includes AlphaFold predictions, but it was unclear whether these are the EMBL-EBI predictions or custom ones? If they are from EMBL-EBI, I believe these use the Uniprot gene models/protein sequences - are these identical to TriTrypDB?

**Response:**

The integrated AlphaFold predictions are the EMBL-EBI predictions, currently covering sequences from the “one sequence per gene” UniProt reference proteome version UniProt 2021_04. These will be automatically updated as AlphaFold makes further releases.

Currently, just over 250,000 of the approximately 350,000 genes with AlphaFold predictions in TriTrypDB have an entry in UniProt. For these genes, the protein sequence in UniProt, and thus the protein sequence used for structural prediction, matches the protein sequence in TriTrypDB exactly. For the remainder of genes we find close matches in UniProt by protein sequence similarity. In these cases, the protein sequence used for the prediction may not exactly match the protein sequence in TriTrypDB. In these cases, we clearly indicate in the AlphaFold table on the gene record page that the UniProt association is made by sequence similarly, however we are currently unable to provide an e-value or similarity score for these associations. We are working with our collaborators at EBI to rectify this. We have added two additional sentences in the manuscript (**page 7**).

**Comment:**
The update schedule is impressive, with the bimonthly updates, and regular version releases. While this versioning is clear, I find it unclear exactly what is versioned in each release, eg. Most genome sequences are identical between TriTrypDB versions, while some gene models and almost all downstream bioinformatic predictions may not. Some guidance on how to stably refer to analyses would be useful, eg. For a statement like "the L. Infantum ortholog of Tb927.4.1310 is LINF_340041500" should it cite the specific TriTrypDB version that was used?
Old versions of gene models are available, are old versions of the wider database?

**Response:**

One can find the last time the genome sequence, gene models or functional annotations updated in TriTrypDB in the release history of the dataset record page of individual genomes (e.g. <https://tritrypdb.org/tritrypdb/app/record/dataset/DS_31282613b1>). It is correct that the genome assembly will remain the same across TriTrypDB releases (unless updated by the actual provider) and structural annotations were only updated in a few releases for even the curated genomes. The list of individual genes updated in a release can be found from the annotation logs associated with the release news of the respective release.

One can download older versions of annotations (GFF/ GAF files) and sequences (fasta files) from the downloads section. In addition, text files containing codon usage and gene aliases and xml files containing NCBI link outs are currently made available under the downloads section. However, old versions of other datasets within the database are not available via downloads. As suggested by the reviewer, it is always a good practice to mention the database version used in methods/ results sections of manuscripts. We have added new text under the subsection on ‘Structural annotation’ (**page 16**).

**Comment:**
On a related note, the community uses gene ID as a stable reference ID, however it is not clear how stable these are. In T. brucei, for example:
Tb927.9.15020: Changed start codon.
How is versioning of such changes shown? I could not find this information, and it could easily trip up reanalysis of older data.
Genes can also be removed without trace, eg:
Tb927.1.3970: Removed gene model.
I understand that it is important to remove dubious genes which lack any information that they are "real", but older transcriptomic and proteomic data will certainly include such dead gene IDs which are hard to back-trace.

**Response:**

The gene IDs remain stable across releases and with updates to gene models such as changed start codons/ CDS etc. The gene IDs are not therefore versioned with gene model updates. Even when gene IDs changed in the past, previous IDs were integrated into gene pages and one can search a gene with previous identifiers. In this regard, we are following standard practice for other large-scale gene/protein databases, where minor changes to gene or protein sequences do not trigger obsoletion or changes to a given identifier.

One can find information about gene model changes from the annotation logs attached to our release news. Although gene model changes are not visible to a user in the gene pages, they have been tracked in our backend curation database. For example, we can revert a gene that was previously made obsolete in our curation database and integrate them in our bimonthly releases. In addition, we encourage the researchers to add the version of the database they used for their data analysis in the methods section of the manuscript. Although a user cannot access a previous database version in the live database, the download section has sequences and annotations from all previous releases and users can therefore use these flat files to make comparisons and conduct analyses, if required (as discussed above for the previous comment). Text has been added to the Structural annotation sub-section of the manuscript to explain these issues (**page 16**).

**Comment:**
The origin of GO term data is unclear - some indication of the workflow of assigning predicted and experimentally shown GO classes would be useful.

**Response:**

We have provided GO annotations using the guidelines specified by the Gene Ontology Consortium. One should look for the metadata displayed with the GO annotations in the gene page tables. They will find the evidence codes (indication of methods used: e.g. sequence similarity, direct assay, mutant phenotypes, automated electronic annotations etc), source (InterPro, GeneDB etc) and reference (PubMed ID, if this is from a published manuscript). An additional paragraph (**pages 18/19**) and a supplementary figure (**S3 Fig**) have been added to describe this.

**Comment:**
Better mobile device support would be very desirable, though I recognise that this would be an enormous investment. I suspect this would be especially for the users in countries directly affected by these diseases where mobile infrastructure is often better. Some minor changes, eg. to gene pages when the left index bar is open, could go a long way.

**Response:**

We agree that the user interface on mobile devices with small screens could be significantly improved, however it is our experience that the majority of users access our databases using laptops, even in countries where mobile infrastructure is the predominant method for accessing the internet. While we do not currently plan to invest effort in improving the user experience on mobile devices, global accessibility is important to us, and we put significant effort into ensuring that our sites remain usable on low-bandwidth connections.

**Comment:**
After making all these criticisms, I would like to finish with a few clear new positives:
Organism preference is a great little feature.
The search pipeline is still very powerful and useful.
The Apollo tool looks powerful and I look forward to trying it out.
Similarly, the single cell seq data visualisation is valuable and will be interesting to explore.
Community integration and annotation of curated genomes are still overall excellent.

**Response:** Many thanks to the reviewers for highlighting these new features.

**Reviewer #3:** The submitted manuscript describes several of the key functions of TriTrypDB, the main resource for functional data mining in Kinetoplastid research. The power of TriTrypDB lies in the fact that it not only serves as a collection of datasets but that it allows the clever integration of hundreds of datasets, thus enabling the easy extraction of relevant information. It is impressive how TriTryp has been able to keep up with the ever-increasing generation of NGS, mass-spectrometry and microscopy data over the last decade and managed to incorporate them into one well-functional database. We are thankful for the many new additions, especially the alpha-fold predictions, tools to visualize sc-RNA-seq data and the incorporation of the TrypTag data.

We also appreciate how the TriTryp team fosters the participation of the community to continually improve the quality of the entries, either by enabling the addition of user comments, by using the integrated curation tool Apollo to correct/add annotations and by offering workshops on how to use the database.

Our lab works with TriTrypDB on a daily basis and is truly thankful for the efforts the authors have put into developing it. In addition, we appreciate the TriTrypDB team listening to user suggestions on how to improve access to the currently available data and to add new features.

I have reviewed the manuscript jointly with my postdoc Raúl Cosentino and both of us would be happy to see the manuscript published as it is, something we have never written before. However, as always there are some suggestions we would like to make.

**Response:** Our sincere gratitude to reviewers (Nicolai and Raúl) for highlighting the key features of TriTrypDB and its utility in their daily research activities. We are delighted to hear that they were happy to see the manuscript published as it is.

Improvements to the database

**Comment:**
•       We were surprised to see that there are 17 genomes lacking any form of annotation. Given that a ‘draft’ genome annotation based on sequence homology can be easily done using tools like Companion, wouldn’t it be worth adding it?

**Response:**

The reviewer raises a good point. Due to the wide ranging development work and dataset integration we do, *de novo* annotation was kept out of our scope and we rely on our data providers for genome annotations. However, it should be noted that the majority of the un-annotated genomes in TriTrypDB are isolates of *Trypanosoma cruzi*. These are difficult to annotate using reference-based methods such as Companion or Liftoff because of the extreme expansion of gene families in this species. We do engage with communities, and we do make exceptions where the community indicates that a particular organism is a strong priority. An example of this is our annotation of the *L. amazonensis* genome with Companion as described in the manuscript (**page 17**). We have expanded this subsection (‘Structural annotation’) further to clarify the points raised by the reviewers.

**Comment:**
•       While the community annotation from Apollo is certainly a great feature, at times it can be confusing/misleading. Looking at a region on chr 2 of the T. b 927 genome in the ‘gene models’ category, we see many annotations from nanopore direct RNA sequencing that do not match any of the genes in the reference, e.g. KS17gene_3200a, KS17gene_3201a. <https://tritrypdb.org/tritrypdb/app/record/gene/Tb927.10.15350#GeneModelGbrowseUrl>
We acknowledge that such annotations are unavoidable with un-curated community annotations, but maybe it would be helpful to clearly list the authors of such annotations. Here the author is listed as api@local.host, which does not tell us much.

**Response:**

We agree with the reviewers that it was not easily visible to the users where these annotations were from and what was the actual evidence behind them from the genome browser view. These non-coding RNA annotations were integrated from Guegen *et al* (PMID:35704590). As these were loaded in bulk using our automated pipelines, the user was provided as [api@local.host](mailto:api@local.host) (This is basically VEuPathDB). However, one could find the actual publication (PubMed ID) in the dbxref section of the gene (see screenshot attached). We agree this is not easily findable given the nature of the Apollo pipeline (an external tool) and we will work on improving these features in collaboration with Apollo core developers. We have also added additional text to the ‘Community annotation via Apollo’ section (**page 21**) to highlight that additional evidence in the form of comments and PubMed IDs can be added on the annotation editor window in order to support their gene model predictions or functional annotations.

The models are scheduled to be integrated in release 64 patch build.

**Comment:**
•       Why are the “Origins of Replication” datasets listed in the category of “Structural variation”? We would associate “Structural variations” with large insertions/deletions or chromosomal rearrangements. Given the transient nature of “Origins of Replication”, maybe they would better fit in the “Epigenomics” category.

**Response:**

Our apologies for this error. In JBrowse, the Origin of Replication tracks are in the “Sequence Analysis” section, which contains non-gene features such as centromeres and repeat regions. We have updated the ontology term associated with these datasets so that the category in the datasets table is consistent with the location in JBrowse (this change will go live in December), and altered the **table 1** in the manuscript to match.

**Comment:**
•       Regarding the “Search strategy system” the authors write, “Saving a strategy retains the order of steps and parameter values, but not the actual results as subsequent database versions containing new data may alter the results”. To increase reproducibility, it would be great if users could store (and share) which search strategy and which specific database they used. This would allow other users to reproduce the results. Such a feature would probably require the option to choose a database version in which to run a search strategy. Maybe it would be useful to implement such an option.

**Response:**

When saving a search strategy, one can add a detailed description, where they will be able to provide all required details such as database version etc. However, one cannot choose the database version currently as we only provide the most recent database version in the live instance. The recent addition of a new feature to store gene lists from search strategies also allow a user to save results (in addition to downloading results). This allows a user to compare the current results from an already saved search strategy with a previously stored gene list. The information on these features were added under the subsection on ‘Search strategy system’ (**page 13**).

**Comment:**
•       Running the big-PI predictor for a gene we received the following error message: “Can't connect to mendel.imp.ac.at:443 (certificate verify failed) SSL connect attempt failed with unknown error error:14090086:SSL routines:ssl3_get_server_certificate:certificate verify failed at /usr/share/perl5/LWP/Protocol/http.pm line 51.”
We are aware that the predictor is an external tool but maybe the authors could check to make sure that this was just a temporary error message.

**Response:** Many thanks to the reviewers for pointing out this issue. It has now been fixed.

**Comment:**
•       Given that the majority of Trypanosome brucei research is done with the lister 427 strain, it would be great if it could be added as an additional curated reference strain.

**Response:**

We appreciate that the Lister 427 strain of *Trypanosoma brucei* is an important genome for the community, and adding this as a reference is a subject of internal discussion. We are currently discussing the loading of the fully phased assembly of this strain published in 2021 with the authors of that paper (PMID:34541528). As there is significant overhead in promoting an assembly to a reference, we will promote the new assembly once it is loaded. This is likely to be a project with multiple phases, which will involve making the new assembly available, transferring curated annotations from the 927 strain (our current reference), a period of community consultation, loading of functional data on this assembly, and loading into Apollo for further community annotation.

Improvements to the manuscript

**Comment:**
•       It would be interesting to see how the number of datasets incorporated into TriTrypDB has increased over the last decade. Would it be possible to plot this? Such a plot would also nicely illustrate to the reader the amount of data available in TriTrypDB.

**Response:** We have added **S1 Fig** with an additional sentence under the Integrated datasets subsection (**pages 4/5**).

**Comment:**
•       In the “Future perspectives” section, it may be worth mentioning the prospect of working with phased diploid genomes and the challenges this could cause.

**Response:**

We thank the reviewers for this suggestion. Many of the challenges associated with phased genomes are related to poor representation of these genomes in INSDC databases. While we are in discussion about this with contacts at NCBI, we have limited insight into their operations and therefore don’t feel empowered to discuss this in significant depth. However, given the importance to this community, we have added a sentence or two to the “Future Perspectives” section (**page 23**).
